# ABO Blood Type and Thromboembolic Complications after Intracerebral Hemorrhage: an exploratory analysis

**DOI:** 10.21203/rs.3.rs-3108135/v1

**Published:** 2023-07-27

**Authors:** Natasha Ironside, Kara Melmed, Ching-Jen Chen, Setareh Omran, Soojin Park, Sachin Agarwal, E. Sander Connolly, Jan Claassen, Eldad A. Hod, David Roh

**Affiliations:** University of Virginia; New York University Grossman School of Medicine; University of Texas Health Science Center at Houston School of Dentistry: The University of Texas Health Science Center at Houston School of Dentistry; Oregon Health & Science University Neurological Sciences Institute: Oregon Health & Science University Brain Institute; Columbia University Medical Center: Columbia University Irving Medical Center; CUMC: Columbia University Irving Medical Center; Columbia University Irving Medical Center; Columbia University Medical Center: Columbia University Irving Medical Center; CUIMC: Columbia University Irving Medical Center; Columbia University Irving Medical Center

**Keywords:** ABO blood type, thromboembolism, intracerebral hemorrhage

## Abstract

**Background and Purpose:**

Non-O blood types are known to be associated with thromboembolic complications (TECs) in population-based studies. TECs are known drivers of morbidity and mortality in intracerebral hemorrhage (ICH) patients, yet the relationships of blood type on TECs in this patient population are unknown. We sought to explore the relationships between ABO blood type and TECs in ICH patients.

**Methods:**

Consecutive adult ICH patients enrolled into a prospective observational cohort study with available ABO blood type data were analyzed. Patients with cancer history, prior thromboembolism, and baseline laboratory evidence of coagulopathy were excluded. The primary exposure variable was blood type (non-O versus O). The primary outcome was composite TEC, defined as pulmonary embolism, deep venous thrombosis, ischemic stroke or myocardial infarction, during the hospital stay. Relationships between blood type, TECs and clinical outcomes were separately assessed using logistic regression models after adjusting for sex, ethnicity and ICH score.

**Results:**

Of 301 ICH patients included for analysis, 44% were non-O blood type. Non-O blood type was associated with higher admission GCS and lower ICH score on baseline comparisons. We identified TECs in 11.6% of our overall patient cohort. Although TECs were identified in 9.9% of non-O blood type patients compared to 13.0% in O blood type patients, we did not identify a significant relationship of non-O blood type with TECs (adjusted OR = 0.776, 95%CI: 0.348–1.733, p = 0.537). The prevalence of specific TECs were also comparable in unadjusted and adjusted analyses between the two cohorts. In additional analyses, we identified that TECs were associated with poor 90-day mRS (adjusted OR = 3.452, 95% CI: 1.001–11.903, p = 0.050). We did not identify relationships between ABO blood type and poor 90-day mRS (adjusted OR = 0.994, 95% CI:0.465–2.128, p = 0.988).

**Conclusions:**

We identified that TECs were associated with worse ICH outcomes. However, we did not identify relationships in ABO blood type and TECs. Further work is required to assess best diagnostic and prophylactic and treatment strategies for TECs to improve ICH outcomes.

## Introduction

Thromboembolic complications (TECs) are preventable causes of in-hospital mortality and morbidity in stroke patients.^1^ Patients with intracerebral hemorrhage (ICH) harbor an elevated risk of venous thromboembolism (VTE), compared to ischemic stroke.^2, 3^ Potential contributing factors include genetic differences, greater functional impairment, and delayed initiation of VTE prophylaxis.^2, 3^ While the Clots in Legs or Stockings after Stroke trials provided evidence for the routine use of intermittent pneumatic compression devices, there is a paucity of prospective data guiding the timing of initiation of VTE chemoprophylaxis after ICH with the goals of maximizing benefits while minimizing the risk of ICH expansion.^4–8^ Similarly, indications for and timing of restarting anticoagulant and/or antiplatelet therapy for prevention of non-VTE related thrombotic complications such as arterial thromboembolism after ICH remains uncertain.^9, 10^

ABO blood type has been implicated in the development of thromboembolic and hemorrhagic disorders.^11–13^ In healthy blood donors, non-O blood type has been associated with an increased risk of TECs, compared with O blood type.^11, 13^ This has been attributed to the higher levels of von Willebrand factor and factor VIII in patients with non-O blood types.^14, 15^ In ICH patients, there appears to be conflicting data regarding how blood type impacts coagulopathy, specifically in regards to hemostasis and ongoing bleeding.^16, 17^ It is unknown whether blood type is a relevant risk factor for coagulopathy and impacts TECs after ICH as has been seen in population based studies. Identification of risk factors associated with TECs after ICH would improve the understanding of disease pathophysiology, allow for improved risk stratification, and permit earlier initiation of treatment or prophylaxis for these deleterious complications. We aimed to assess whether ABO blood type is associated with TECs after ICH. We separately assessed relationships of ABO blood type and TECs with ICH outcome.

## Methods

### Study design

Between February 2009 and November 2018, consecutive patients presenting with spontaneous ICH at our institution were prospectively enrolled in the Intracerebral Hemorrhage Outcomes Project (ICHOP). The ICHOP study methods have been previously described in detail.^18^ The study was approved by the Institutional Review Board committee at our institution, and written informed consent was obtained from all patients (or respective legal guardians when the patient was unable to provide consent) participating in the study. The clinical care of all participants was managed according to American Heart Association Guidelines.^19^ Participants or designated proxies underwent a standardized data collection protocol, which included a personal interview and medical chart abstraction. This study follows the guidelines set forth by the Strengthening the Reporting of Observational Studies in Epidemiology statement.^20^

### Patient identification and selection

ICH patients were diagnosed via admission head computed tomography (CT). The inclusion criteria for this study were: (1) age ≥18 years (2) available admission blood type and routine laboratory studies data, and (3) available in-hospital thromboembolic complications data. The exclusion criteria for the study were: (1) primary intraventricular hemorrhage and those with ICH related to trauma, brain tumor, hemorrhagic transformation of an ischemic cerebral infarct, vascular malformation or any other suspected secondary causes (2) early withdrawal of care, which was defined as within 24 hours of admission, (3) prior history of malignancy, (4) prior history of VTE or systemic emboli, (5) prior anticoagulant use history, (6) laboratory evidence of coagulopathy on admission, defined as PT > 20s or PTT > 50s, or (7) laboratory evidence of thrombocytopenia on admission, defined as platelet count < 50 × 10^9^/ L.

### Clinical data

Baseline demographic and medical history data included age, sex, ethnicity, history of atrial fibrillation, coronary artery disease, congestive heart failure, hyperlipidemia, hypertension, and diabetes mellitus. Medication history data included anticoagulant, antihypertensive, and antiplatelet use prior to admission. Clinical and laboratory data included systolic blood pressure, blood glucose, platelet count, international normalized ratio, partial thromboplastin time, National Institutes of Health Stroke Scale, and Glasgow coma scale score on admission.^21^ Radiographic characteristics included ICH volume, ICH location (categorized as lobar, basal ganglia/thalamus, brainstem, or cerebellar), and presence of intraventricular hemorrhage. Pre-morbid functional status was measured by the modified Rankin Scale (mRS).^22^

Intermittent pneumatic compression devices were initiated on admission for VTE prophylaxis in all patients. Prophylactic dosing of subcutaneous unfractionated heparin was routinely initiated at 24 to 48 hours after admission if (1) hematoma stability had been demonstrated on the 24-hour CT scan, and (2) craniotomy/craniectomy for decompression or hematoma evacuation was not performed.

### ABO blood type

ABO and Rhesus blood types were obtained for all ICH patients as part of our local clinical protocol upon admission in anticipation for any transfusion therapy need.^16^ Blood type was defined as a binary exposure variable (Non-O vs O) given previously reported increased prevalence of TECs in Non-O (i.e., type A, type B, type AB) patients.^16^

### Outcomes

The primary outcome was composite TEC, comprising pulmonary embolism (PE), deep venous thrombosis (DVT), ischemic stroke, and myocardial infarction (MI) during the ICH hospitalization. These complications were prospectively adjudicated through weekly meetings of study coordinators and clinical team members in conjunction with assessing ICD-9 and 10 diagnostic codes (410, 434, 453, 415). In brief, MI was defined as an elevation of cardiac troponin above the upper reference limit with one or more of the following: symptoms of myocardial ischemia, new ischemic EKG changes, imaging evidence of new regional wall motion abnormality or identification of a coronary thrombus on angiography.^23^ Ischemic stroke was defined as a sudden onset of focal neurologic deficit persisting > 24 hours, associated with neuroimaging evidence of infarction.^24^ DVT was defined as any identified thrombus of the deep venous system on lower or upper extremity doppler ultrasonography. PE was defined as any identified thrombus of the pulmonary arterial system on computed tomography pulmonary angiogram. TECs were separately assessed as venous and arterial TECs.

Clinical outcomes including mortality and poor mRS (mRS 4–6) scores at discharge and 90 days were assessed as secondary clinical outcomes of interest. Functional outcome assessments utilized standardized questionnaires that were administered by trained study staff to participants or their legal representatives via in-person or telephone-based interviews at the time of hospital discharge and at 90 days as previously reported.^18, 25, 26^

### Statistical Analysis

All statistical analyses were performed using the Stata software (version 16.1; StataCorp; College Station, TX). Intergroup baseline differences were reported for non-O vs. O blood type cohorts using Pearson’s chi-squared or Fisher’s exact tests for categorical variables and Student’s *t* or Mann-Whitney U tests for continuous variables, as appropriate. Relationships between blood type, TEC and clinical outcomes were separately assessed adjusted logistic regression models adjusting for sex and race and ICH score. Statistical significance was defined as p < 0.05, and all tests were two-tailed.

## Results

**Figure 1:**
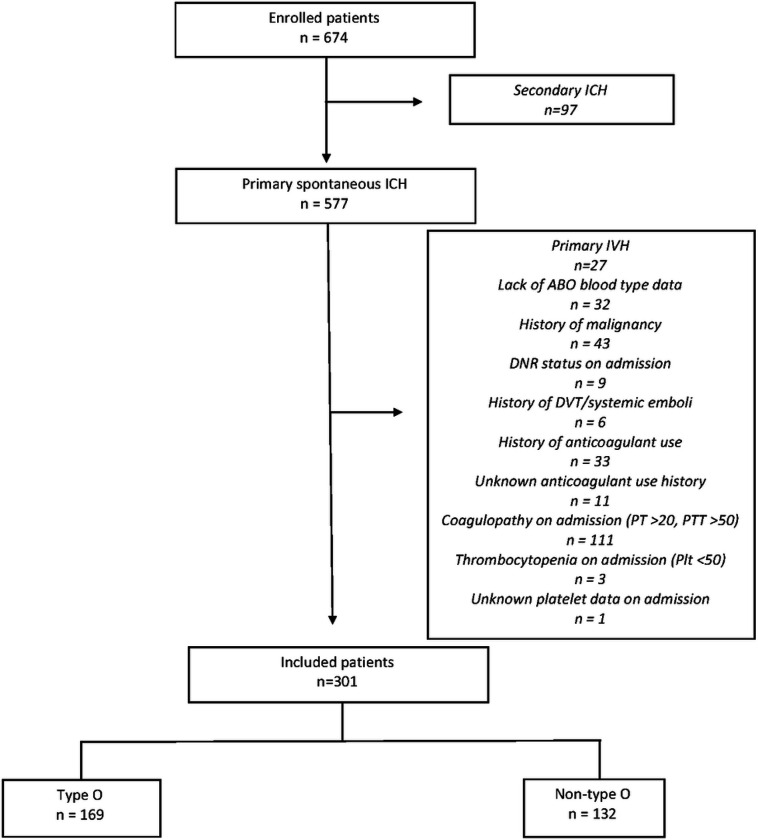
Flow chart of the patient selection process. Abbreviations: ICH = intracerebral hemorrhage, IVH = intraventricular hemorrhage, DNR = do not resuscitation, DVT = deep venous thrombosis, PT = prothrombin time, PTT = partial thromboplastin time, Plt = platelet count.

We identified 301 spontaneous ICH patients meeting criteria for inclusion (Fig. 1). The mean age was 63.8±15.9 years and 47.5% of the overall cohort was female. There were 132 (43.9%) ICH patients who were non-O blood type. Amongst these non-O blood type ICH patients, type A comprised 65.9% (n = 87), type B comprised 28.8% (n = 38), and type AB comprised 5.3% (n = 7). Baseline characteristic differences between ICH patients with non-O blood type and O blood type are listed in Table 1. We identified that patients with non-O blood types appeared to have less severe ICH measured by lower ICH scores and higher admission GCS compared to patients with type O blood. No other differences in baseline ICH volume or other baseline clinical or laboratory characteristics were noted.

We identified 35/301 (11.6%) patients who encountered a TEC during their hospitalization (Table 2). TECs were comprised primarily of arterial events and myocardial infarction accounted for the greatest proportion of arterial TECs. When assessing relationships of ABO blood type and TECs, we did not identify an association of non-O blood type with composite TECs (aOR = 0.776 [0.348–1.733], p = 0.537). Separate analyses similarly did not find any association of blood type with venous or arterial TEC.

When assessing relationships of TEC as the exposure on clinical outcomes, we identified that TECs were associated with increased odds of poor mRS at 90 days (aOR = 3.452 [1.001–11.903], p = 0.050). However, we did not identify relationships of ABO blood type as the exposure on poor 90-day mRS (aOR = 1.006 [0.470–2.150], p = 0.988).

## Discussion

In spontaneous ICH patients, the in-hospital incidence of TECs is reported to range from 3–22% and the risk of VTE is up to four times higher than in patients with acute ischemic stroke.^2, 8, 27, 28^ Though chemical thromboprophylaxis has been recommended in ICH patients to prevent these complications, TECs continue to occur, necessitating improved risk stratification strategies for prevention and guidelines for timing of treatment initiation.^8^ While ABO blood type has been implicated as a risk factor for coagulopathy and thromboembolism, we did not identify a relationship between ABO blood type, specifically non-O blood types, with TECs in our racially diverse cohort of ICH patients.^11–13^ Although ABO blood type did not appear to impact clinical outcomes, we did identify that TEC were independently associated with worse clinical outcomes among ICH patients.

In healthy population studies, non-O blood phenotypes (i.e., A, B, AB) have been identified as a risk factor for both venous and arterial thromboembolism.^11, 13, 29^. This has been posited to occur due to elevated concentrations of factor VIII and von Willebrand factor in individuals with non-O blood, interactions between the immune-dominant blood group antigens in the endothelial lining of blood vessels and von Willebrand factor, and genetic differences in serum levels of soluble intracellular adhesion molecule 1, tumor necrosis factor, and soluble E- and P-selectin.^14, 15, 30–32^ Conversely, O blood type has also been established as a risk factor for bleeding.^12, 33^ In a meta-analysis of 22 studies, comprising 466,752 patients, Dentali et al. reported a significantly higher prevalence of O blood phenotype in patients with bleeding complications compared to those without bleeding complications.^33^

While these relationships have been observed in non-brain injured patients, the impact of ABO blood type on coagulation/coagulopathy after ICH is less clear.^12, 34^ Inconsistent relationships of different blood types on hematoma expansion after ICH have been reported, ^16 17^ but there have not been any reports to date assessing the impact of ABO blood type on TECs. Though our study was limited by its small sample size and single center, retrospective design requiring replication in a larger dataset, it provides initial data to suggest that ABO blood type may not confer the same risk of TECs in active bleeding disease states compared to healthy populations. Paradoxically, we observed an increased number of TECs in type O blood type patients, although this finding did not reach statistical significance. Though speculative, it is possible that the known acute inflammatory state following ICH alters systemic coagulation cascade activation which may overcome any relevant baseline coagulation differences that may be present between different ABO blood groups during non-disease states.^35, 36^ Thus, further study is necessary to understand the complex thromboinflammation that occurs following ICH and its impact on downstream bleeding and thrombotic complications.

In regards to the impact of blood type on ICH outcomes, our findings are consistent with prior studies which did not observe relationships between ABO blood type and functional outcome after ICH.^16, 36, 37^ We did, however, find independent associations between TECs and poor 90-day functional outcome after ICH. This is congruent with previously reported associations between VTE and worse functional outcome at discharge, 3 months and one year.^2 28^ The management of TECs presents a challenge in acute ICH, due to the competing interests of simultaneously preventing intracranial hematoma expansion and systemic thromboembolism. In a retrospective study of 42 ICH patients with TECs, therapeutic anticoagulation was initiated at the discretion of the treating physician, on average 13 days after the ictus, and resulted in hemorrhage expansion in one patient.^38^ IVC filter use, which has not been well studied in ICH patients, was not associated with differences in overall mortality in a randomized clinical trial of critical care patients with significant bleeding risk.^39^ Taken together, the findings from the present study emphasize the importance of TEC screening and prevention in ICH patients. Future prospective studies should seek to clarify appropriate timing of chemoprophylaxis initiation, determine safety of therapeutic anticoagulation, and identify novel methods for risk stratification and treatment.

While the relatively uniform VTE prophylactic practice and multidisciplinary adjudication of complications were a strength of this study, there are several limitations worth highlighting. As aforementioned, our primary limitation was our relatively small, single-center cohort size. It is possible that our exploratory analysis was underpowered and would have identified significant associations of blood type with TECs if a larger cohort had been evaluated. Similarly, though our ICH cohort was a racially diverse patient population, it is unclear whether our findings would be generalizable to other ICH patient cohorts as race/ethnicity and geographic locations also have known associations with blood type and coagulation. Additionally, given our small sample size, we were unable to evaluate individual effects of the A, B or AB blood types on our outcomes of interest. Furthermore, because the ICHOP study was not designed to investigate relationships between ABO blood type and TECs after spontaneous ICH, the present analysis is subject to confirmation bias in that variables were chosen based upon data availability. In this regard, our TEC outcomes were only identified based on clinically obtained diagnostic testing. While this reflects clinical practice, it is unclear what the true prevalence of VTE would be in this patient population if systemically screened. Finally, our study did not have comprehensive available coagulation parameters of interest including von Willebrand factor and factor VIII to be able to establish the absence (or presence) of relevant coagulation differences between non-O and O blood ICH patients. Thus, further work assessing coagulation factors and their impacts on ICH outcomes will be necessary to better understand specific drivers behind hemostasis and thrombotic complications in this vulnerable patient population.

## Conclusion

ABO blood phenotype was not associated with TECs or clinical outcome after spontaneous ICH. TECs were associated with worse functional outcome at 90 days. Further work is required to assess best diagnostic and prophylactic and treatment strategies for TECs to improve ICH outcomes.

## Figures and Tables

**Table 1:** Comparison of baseline characteristics between ICH patients with blood type O and non-type O phenotypes.

Baseline Characteristics	Overall cohort (n = 301)	Non-type O (n = 132)	Type O (n = 169)	Value
Age, mean years (SD)	63.8 (15.9)	63.0 (14.9)	64.5 (16.7)	0.420
Female, n (%)	143 (47.5)	63 (47.7)	80 (47.3)	0.946
Race, n (%)				0.798
White	67 (23.1)	26 (20.5)	41 (25.2)	
Black	93 (32.1)	44 (34.7)	49 (30.1)	
Asian	17 (5.9)	8 (6.3)	9 (5.5)	
Hispanic	11 (38.6)	49 (38.6)	63 (38.7)	
Other	1 (0.3)	0 (0)	1 (0.6)	
**Medical history**				
Myocardial infarction, n (%)	13 (4.3)	7 (5.3)	6 (3.6)	0.458
Coronary artery disease, n (%)	33 (11.0)	17 (12.9)	16 (9.5)	0.347
Ischemic stroke, n (%)	26 (8.6)	11 (8.3)	15 (8.9)	0.868
Diabetes, n (%)	79 (26.3)	35 (26.5)	44 (26.0)	0.925
Hypertension, n (%)	237 (78.7)	104 (78.8)	133 (78.7)	0.985
Hypercholesteremia, n (%)	78 (25.9)	37 (28.0)	41 (24.3)	0.459
**Medication use**				
Antiplatelet, n (%)	99 (32.9)	43 (32.6)	56 (33.1)	0.918
**Laboratory tests**				
PT, mean sec (SD)	13.5 (1.5)	13.5 (1.5)	13.5 (1.6)	1.000
PTT, mean sec (SD)	28.9 (4.2)	28.8 (4.3)	28.9 (4.1)	0.821
INR, mean (SD)	1.1 (0.2)	1.1 (0.1)	1.1 (0.2)	0.790
Platelets, mean ×10^3^/uL (SD)	225.9 (74.0)	228.5 (76.3)	223.9 (72.2)	0.595
Rhesus positive, n (%)	282 (92.3)	126 (95.5)	156 (92.3)	0.265
**Clinical/Radiographic findings**				
Baseline mRS score, median (IQR)	0 (0–1.5)	0 (0–1)	0 (0–2)	0.894
Admission GCS, median (IQR)	11 (7–15)	13 (8–15)	10 (6–14)	0.009
ICH volume, median mL (IQR)	35.4 (4.938.3)	33.4 (5–32.1)	36.7 (41.4–45)	0.372†
Deep ICH location, n (%)	169 (64.3)	71 (62.8)	98 (65.3)	0.675
Lobar ICH location, n (%)	94 (35.7)	42 (37.2)	52 (34.7)	0.675
Brainstem ICH location, n (%)	13 (4.9)	3 (2.7)	10 (6.7)	0.137
Infratentorial ICH location, n (%)	31 (11.8)	13 (11.5)	18 (12.0)	0.902
IVH, n (%)	161 (53.7)	64 (48.5)	97 (57.7)	0.111
ICH score, median (IQR)	2 (1–3)	2 (1–2)	2 (1–3)	0.010

† Indicates log transformed variable.

**Table 2:** Comparison of TEC between patients with blood type O and non-type O phenotype.

			Model 1§		Model 2*	
	Non-type O (n = 132)	Type O (n = 169)	OR [95% CI]	value	OR [95% CI]	Value
Thromboembolic complication, n (%)†	13 (9.9)	22 (13.0)	0.728 (0.353–1.511)	0.396	0.776 (0.348–1.733)	0.537
Arterial embolism, n (%)	7 (5.3)	13 (7.7)	0.672 (0.260–1.736)	0.411	0.727 (0.2741.927)	0.521
Venous thromboembolism, n (%)	6 (4.6)	7 (4.1)	1.103 (0.361–3.356)	0.864	1.368 (0.342–5.464)	0.658
Poor discharge mRS score, n (%)	95 (72.5)	132 (78.6)	0.720 (0.423–1.225)	0.226	1.383 (0.645–2.967)	0.405
Poor 90-day mRS score, n (%)	51 (56.7)	87 (67.4)	0.631 (0.361–1.101)	0.105	1.006 (0.470–2.150)	0.988
90-day mortality, n (%)	24 (26.7)	56 (43.4)	0.474 (0.265–0.848)	**0.012**	0.906 (0.506–1.621)	0.739

§ Unadjusted

* Adjusted for sex, race, and ICH score

† 22/35 (62.9%) patients experienced more than one TEC event during their hospital stay.

Association between TEC and clinical outcomes.

**Table 3:** Association between TEC and clinical outcomes.

			Model 1§		Model 2*	
	TEC (n = 35)	No TEC (n = 266)	OR [95% CI]	p-value	OR [95% CI]	p-value
Poor discharge mRS score, n (%)	31 (88.6)	196 (74.2)	2.689 (0.916–7.895)	0.072	2.987 (0.69912.760)	0.140
Poor 90-day mRS score, n (%)	23 (82.1)	115 (60.2)	3.040 (1.108–8.343)	0.031	3.452 (1.00111.903)	0.050
90-day mortality, n (%)	10 (35.7)	70 (36.7)	0.960 (0.420–2.196)	0.924	0.888 (0.3082.560)	0.826

§ Unadjusted

* Adjusted for sex, race, and ICH score
